# Supplementary figures and images for: Sex-biased genetic programs in liver metabolism and liver fibrosis are controlled by EZH1 and EZH2
Source: PLoS Genet. 2020 May 19;16(5):e1008796. doi: 10.1371/journal.pgen.1008796 (PMC7263639; doi:10.1371/journal.pgen.1008796)

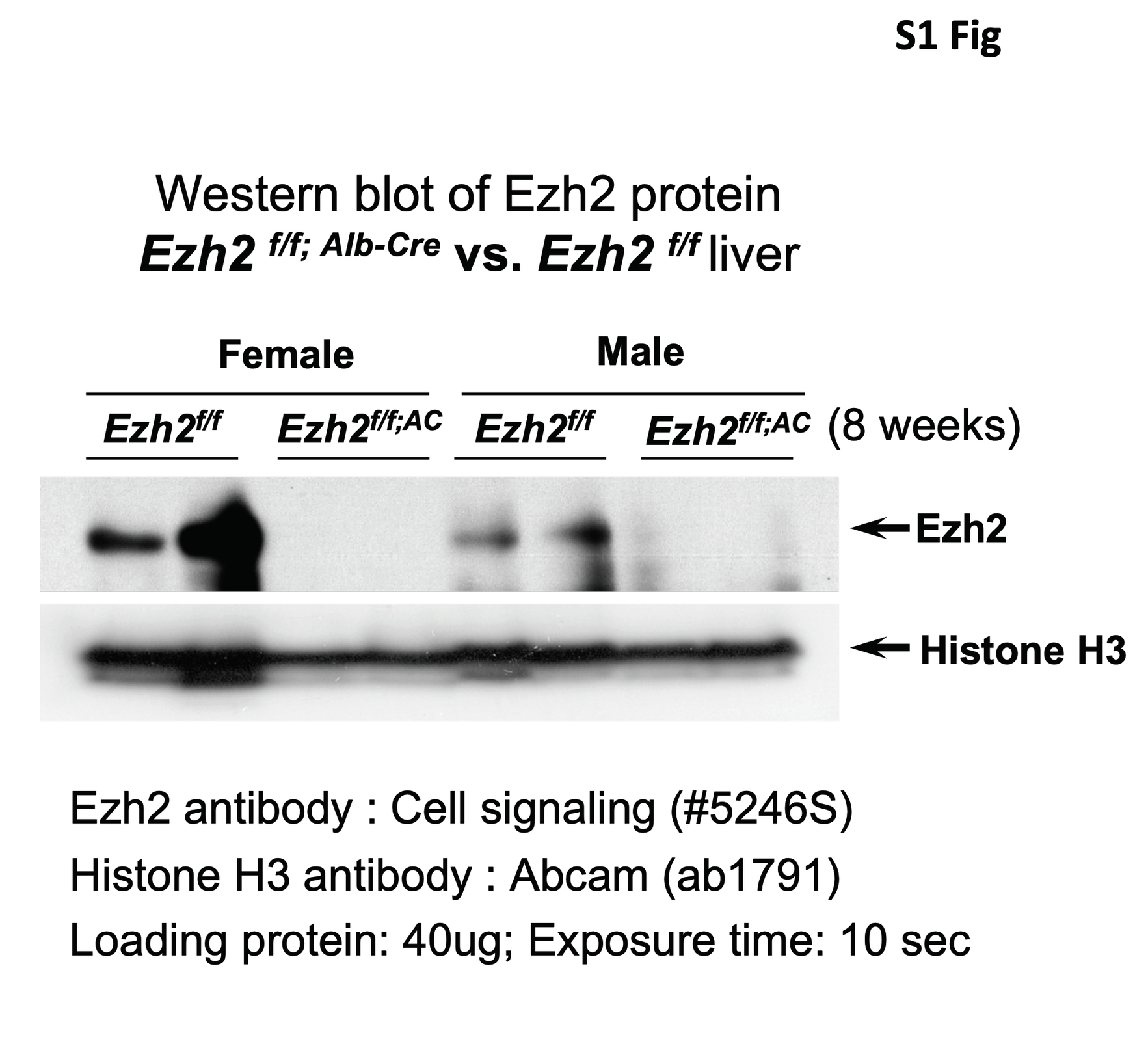

Supplement: S1 Fig — (TIF) [file pgen.1008796.s001.tif]

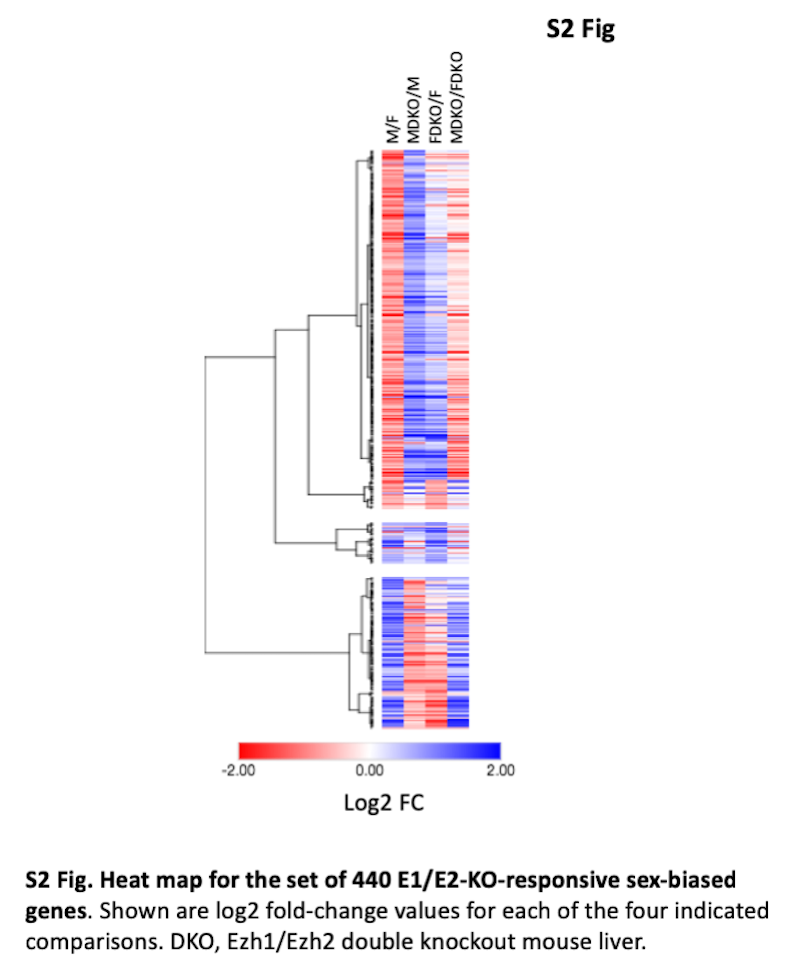

Supplement: S2 Fig — Shown are log2 fold-change values for each of the four indicated comparisons. Decrease in color intensity for many genes (column 4 vs. column 1) indicates loss of sex bias. DKO, Ezh1/Ezh2 double knockout mouse liver. (TIF) [file pgen.1008796.s002.tif]

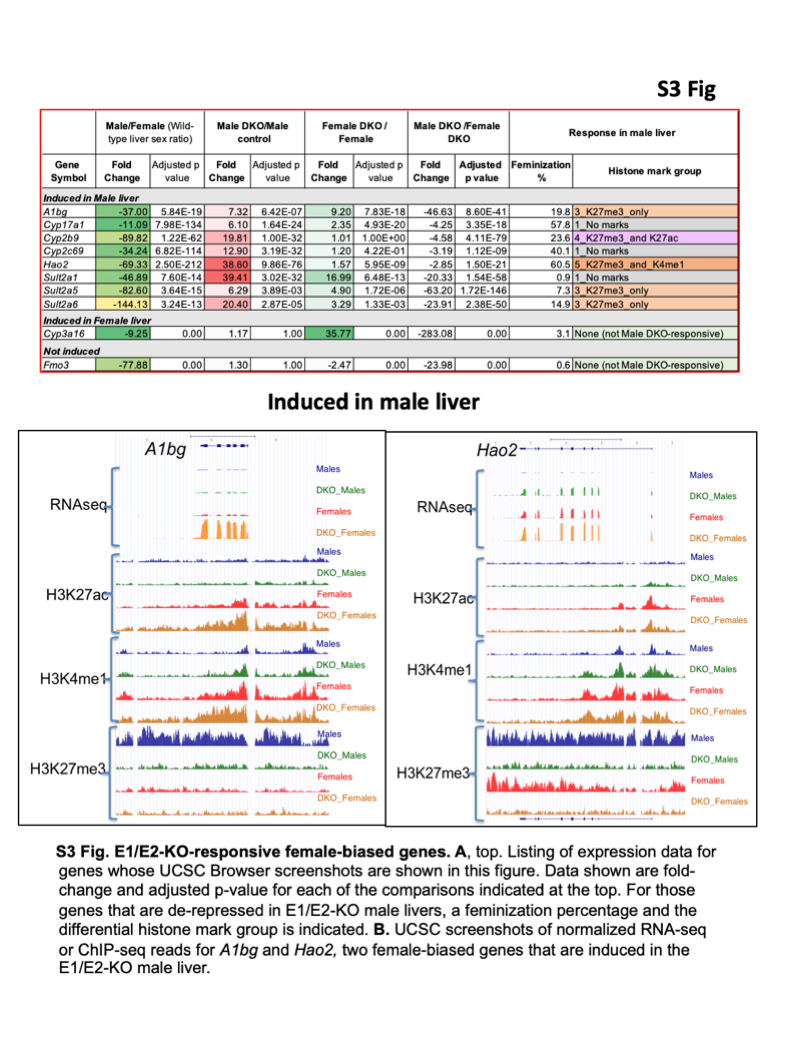

Supplement: S3 Fig — A, top. Listing of expression data for genes whose UCSC Browser screenshots are shown in this figure. Data shown are fold-change and adjusted p-value for each of the comparisons indicated at the top. For those genes that are de-repressed in E1/E2-KO male livers, a feminization percentage and the differential histone mark group is indicated. B. UCSC screenshots of normalized RNA-seq or ChIP-seq reads for A1bg and Hao2, two female-biased genes that are induced in the E1/E2-KO male liver. (TIF) [file pgen.1008796.s003.tif]

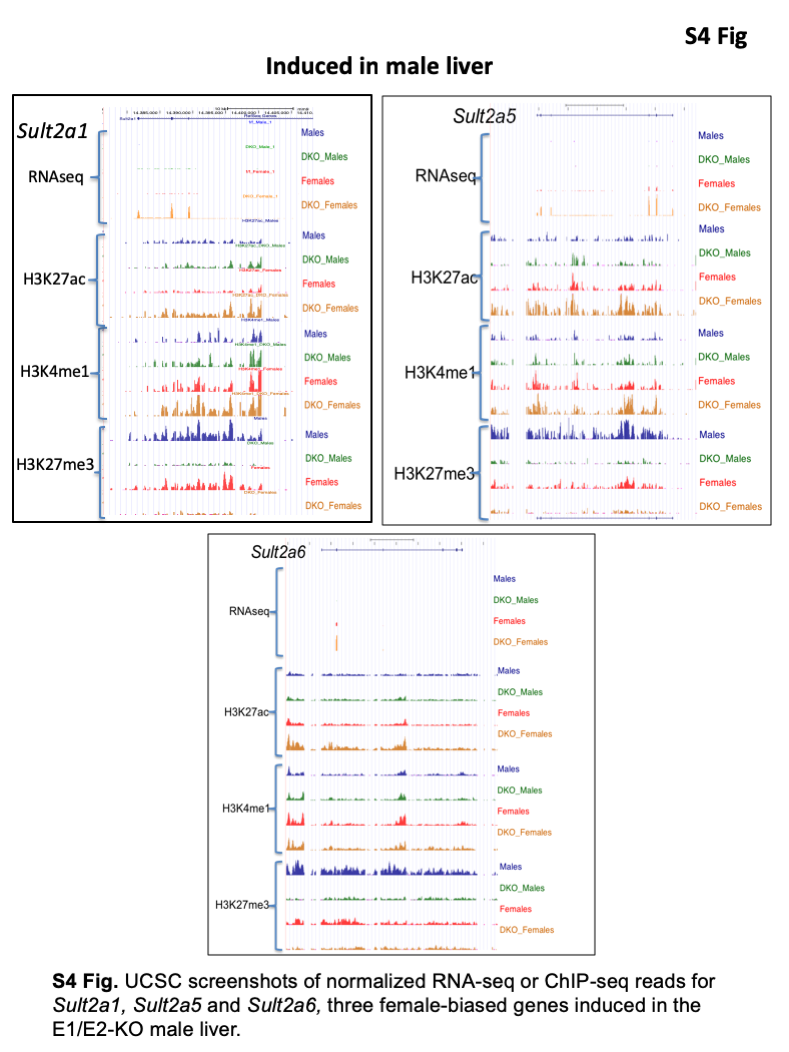

Supplement: S4 Fig — (TIF) [file pgen.1008796.s004.tif]

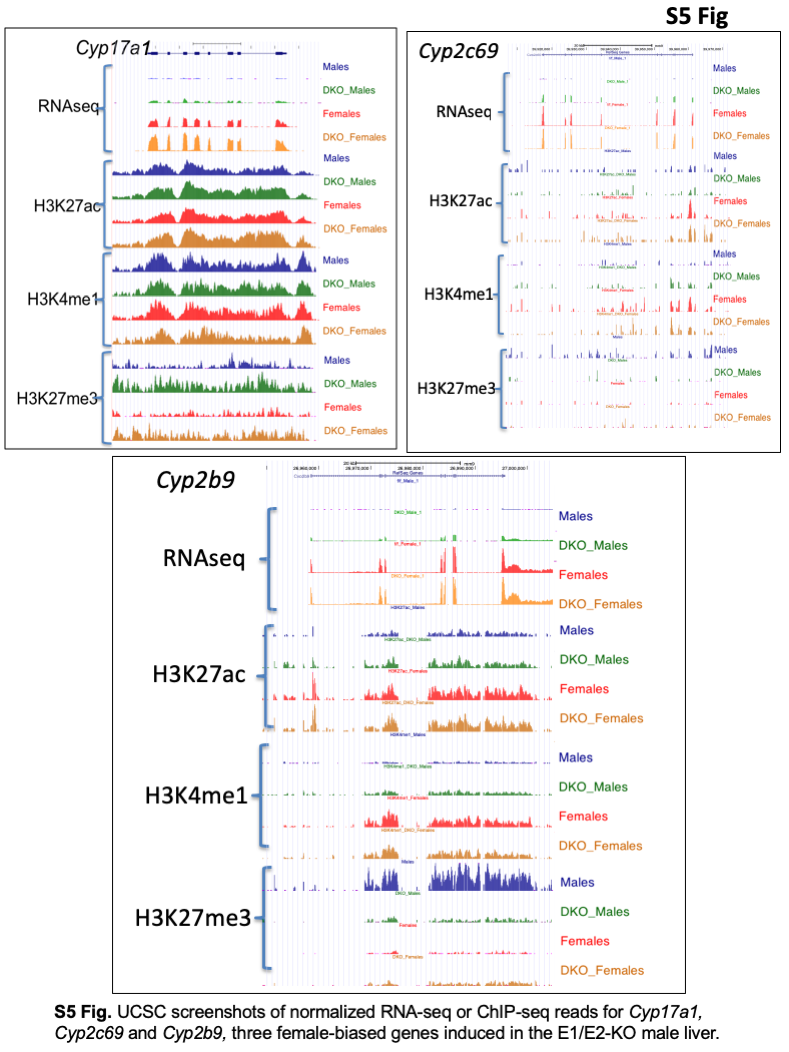

Supplement: S5 Fig — (TIF) [file pgen.1008796.s005.tif]

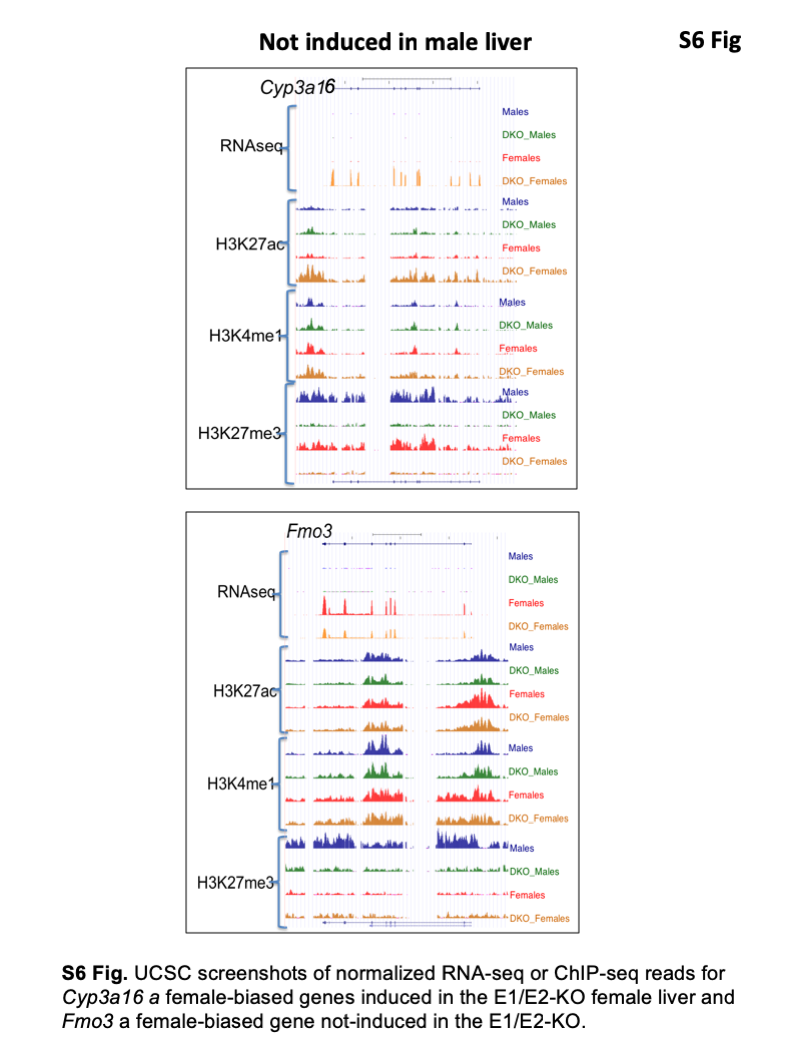

Supplement: S6 Fig — (TIF) [file pgen.1008796.s006.tif]

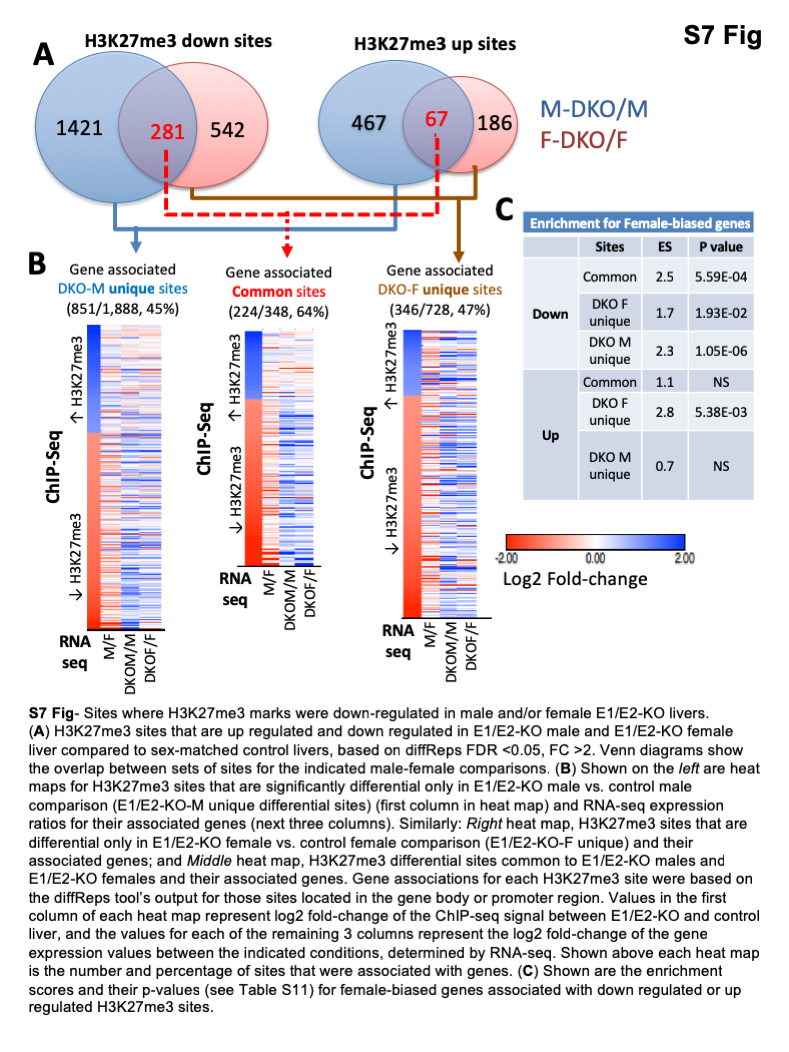

Supplement: S7 Fig — (A) H3K27me3 sites that are up-regulated and down-regulated in E1/E2-KO male and E1/E2-KO female liver compared to sex-matched control livers, based on diffReps FDR <0.05, FC >2. Venn diagrams show the overlap between sets of sites for the indicated male-female comparisons. (B) Shown on the left are heat maps for H3K27me3 sites that are significantly differential only in E1/E2-KO male vs. control male comparison (E1/E2-KO-M unique differential sites) (first column in heat map) and RNA-seq expression ratios for their associated genes (next three columns). Similarly: Right heat map, H3K27me3 sites that are differential only in E1/E2-KO female vs. control female comparison (E1/E2-KO-F unique) and their associated genes; and Middle heat map, H3K27me3 differential sites common to E1/E2-KO males and E1/E2-KO females and their associated genes. Gene associations for each H3K27me3 site were based on the diffReps tool’s output for those sites located in the gene body or promoter region. Values in the first column of each heat map represent log2 fold-change of the ChIP-seq signal between E1/E2-KO and control liver, and the values for each of the remaining 3 columns represent the log2 fold-change of the gene expression values between the indicated conditions, determined by RNA-seq. Shown above each heat map is the number and percentage of sites that were associated with genes. (C) Shown are the enrichment scores and their p-values (see S12 Table) for female-biased genes associated with down-regulated or up-regulated H3K27me3 sites. (TIF) [file pgen.1008796.s007.tif]

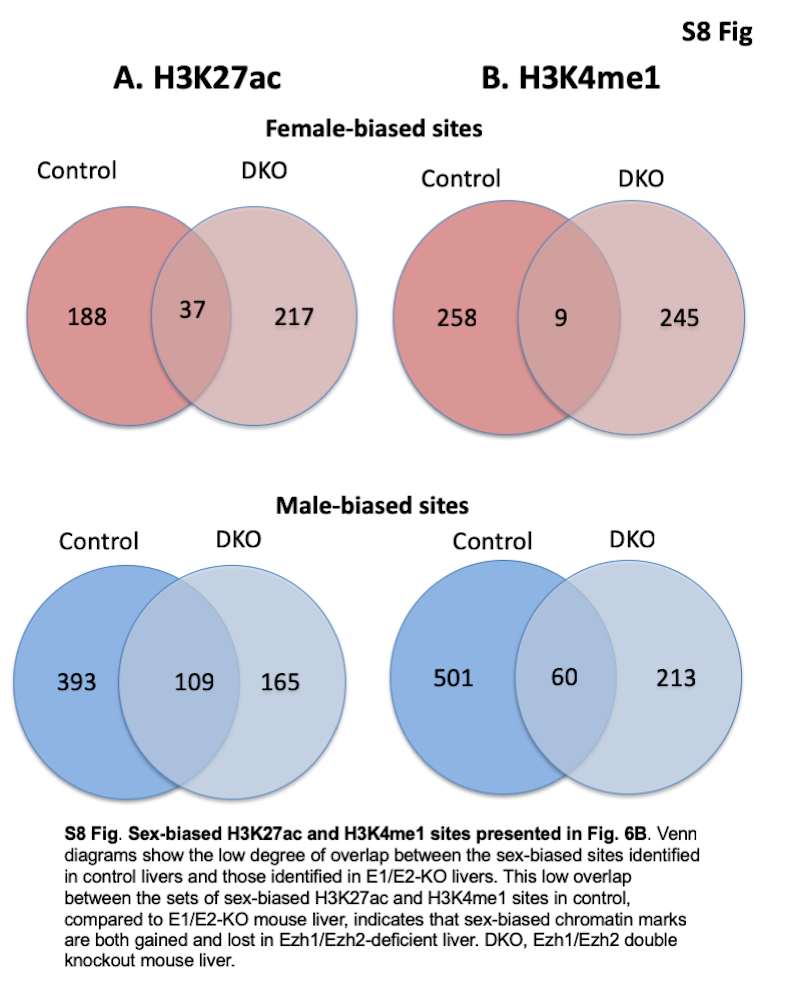

Supplement: S8 Fig — Venn diagrams show the low degree of overlap between the sex-biased sites identified in control livers and those identified in E1/E2-KO livers. This low overlap between the sets of sex-biased H3K27ac and H3K4me1 sites in control, compared to E1/E2-KO mouse liver, indicates that sex-biased chromatin marks are both gained and lost in Ezh1/Ezh2-deficient liver. DKO, Ezh1/Ezh2 double knockout mouse liver. (TIF) [file pgen.1008796.s008.tif]
