# Supplementary material for: Sex-biased genetic programs in liver metabolism and liver fibrosis are controlled by EZH1 and EZH2
Source: PLoS Genet. 2020 May 19;16(5):e1008796. doi: 10.1371/journal.pgen.1008796 (PMC7263639; doi:10.1371/journal.pgen.1008796)
Supplement: Attachment — Submitted filename: PGENETICS-D-19-01963_comments.docx [file pgen.1008796.s021.docx]

**Summary**

The sexual dimorphic hepatic gene expression profile is linked to the sex-biased incidence and severity of liver diseases, such as liver fibrosis and HCC. This manuscript probed an essential role of EZH1 and EZH2, the catalytic components of the polycomb repressive complex-2 (PRC2), for their regulation of the sex-based repressive chromatin histone mark H3K27me3 and the metabolic and fibrosis gene expressions profile in the liver.

This study established that Ezh1/2-based repression of female-biased genes is a major epigenetic mechanism that controls sex-biased gene expression in the male liver and represented a direct demonstration of the functional importance of the sex-biased repressive histone mark H3K27me3.

The author provided comprehensive characterization of the hepatic chromatin modification states such as H3K27me3, H3K27ac and H3K4me1, in combination with the previously published datasets from hypophysectomy and continuous GH infusion models. The up regulation of the genes linked to liver fibrosis and HCC is more significant in the male liver but to a lesser extent in the female liver, which may indicate the clinical relevance of Ezh1/2 in patients with liver disease, however, the functional evidence of this observation *in vivo* is weak.

The study design was clear and straight-forward and the manuscript was well polished, but there are some concerns for which additional experiments and revision of the manuscript should be considered.

**Major points:**

1. Since (i) deletion of Ezh2 in embryonic liver impairs hepatic progenitor expansion, thus the liver differentiation and maturation (ref 25), (ii) Alb-Cre transgene is also active in fetal and neonatal mice (Ref 63), (iii) a more recent study reported that disruption of Ezh1 and Ezh2 in livers caused perinatal hepatocytes to differentiate prematurely (Grindheim et al, Gastroenterology, 156:1834-1848, 2019), whether the combined hepatic Ezh1/Ezh2 deficiency model in this study have similar issue? If so, is this defect also sex-specific? And whether the sex-specific differences in the adult mice of current study are secondary to this developmental defect? And how is this defect linked to the context of sex-specific growth hormone regulation?

2. The author showed the Ezh1/2-dependent and sex-biased regulation of liver fibrosis and HCC-related gene expression in Fig. 7. However, this is largely based on the gene expression analysis, further functional evidence is necessary for the author to draw a solid conclusion. As previous reports mainly used the male mice in their studies (Ref 33 and Grindheim et al, Gastroenterology, 156:1834-1848, 2019), a comparative characterization of the liver fibrosis and damage phenotype between male and female would be informative at this point, either in the older hepatic Ezh1/Ezh2 deficiency mice or in the CCl4-induced hepatotoxity model.

**Minor points:**

3. Fig.2 and 3 are very busy - need to be reorganized or streamlined to make them easier to follow.

4. Fig.3 - To what extent does EZH1/2-dependnent liver chromatin repression contribute to the GH-based regulation on the sex-based gene expression profile? Please clarify in the text.

5. Regarding Discussion section (line 503) - the incomplete loss of H3K27me3 marks at many genomic loci, are there any other additional genes or enzymes that could catalyze the H3K27me3 mark formation? Since Alb-Cre transgene is also active in fetal and neonatal mice (Ref 63).
