# Supplementary material for: Sex-biased genetic programs in liver metabolism and liver fibrosis are controlled by EZH1 and EZH2
Source: PLoS Genet. 2020 May 19;16(5):e1008796. doi: 10.1371/journal.pgen.1008796 (PMC7263639; doi:10.1371/journal.pgen.1008796)
Supplement: Attachment — Submitted filename: Author Responses to Reviewers_March 29.docx [file pgen.1008796.s022.docx]

**Author Responses to Reviewer Comments**

**Reviewer #1**

In this manuscript, the authors investigated the functional impact of sex differences in histone protein modifications related to sex-biased liver diseases. The work was done using mouse models deficient in two enzymes, Ezh1/Ezh2, which form the histone repressive mark H3K27me3. Through RNA-seq, ChIP, and qPCR analyses as well as bioinformatics approaches, the authors found that loss of hepatic Ezh1/Ezh2 disrupts sex-biased H3K27me3 repressive marks, and abolishes the control of sex-biased genes with increased expression in male mouse liver for many female-biased genes and decreased expression of male-biased genes, including those that are involved in liver fibrosis and hepatocellular carcinoma (HCC). This information is interesting and informative to the research community. The manuscript was well written. Most of the data are of high quality.

The following concerns need to be addressed:

1. Based on the qPCR data, the expression of Ezh1 and Ezh2 between male and female were not much difference. If Ezh1/Ezh2 indeed control sex-biased difference in hepatic gene expression between male and female, please address why there is no difference in hepatic Ezh1 and Ezh2 expression between male and female? And also, expression levels of Ezh1 and Ezh2 proteins need to be examined, as mRNA and protein levels are not consistent in many cases.

Author response: Published work, including our own, shows that H3K27 trimethylation occurs in both male and female livers. Further, we show here that the level of H3K27me3 marks is not significantly different in male vs. female liver at 97.4% of genomic loci with H3K27me3 marks (only 549 out of 21,036 SICER-discovered H3K27me3 peak regions identified in male and female liver are sex-biased). Consequently, there is no reason to expect that Ezh1/Ezh2 RNA or protein levels should be different between male and female liver. Rather, our study establishes that H3K27 trimethylation differs between the sexes at a subset of all H3K27me3 sites, regulated by growth hormone, and need not invoke by any sex differences in Ezh1/Ezh2 expression. This point is now made explicit in the revise Results text (lines **281-282**) and in the Discussion on lines **453-458**. Further, we have now added a Western blot confirming that Ezh2 protein is expressed at similar levels in male and female livers, and that the protein is ablated in livers of Ezh2f/f-Alb-Cre mice (lines **135-137; Fig. S1**).

2. There are significant differences in gene expression and pathogenesis in related to hepatic fibrosis and HCC between human and mouse. To translate the findings from the animals, validation of Ezh1/Ezh2-controlled, sex-biased H3K27me3 repressive marks with human liver samples or genomic data is required.

Author response: Yes, there are differences in hepatic fibrosis and HCC between mice and humans. While we think it likely that H3K27 trimethylation plays a role in human liver disease, we do not have access to the human liver samples that would be needed to address that question directly. Accordingly, we did not make claims that our findings are directly translatable to sex differences in human liver, or to hepatic fibrosis and HCC in humans. However, in response to the Reviewer’s comment, we have now carried out two new analyses of published data of human liver samples. First, we examined a set of 121 mouse orthologs of human genes that are up-regulated in advanced liver fibrosis [Grindheim et.al 2019 (PMID:30689973)], and found that 28 (23%) of these genes are responsive in Ezh1/2-DKO in our mouse model (24 up-regulated and 1 down regulated genes), of which 5 sex-biased DKO-responsive genes (including 3 female-biased genes up-regulated in male liver). Genes up regulated in common between advanced human liver fibrosis and in Ezh1/2-DKO mouse liver include biomarkers of liver disease (e.g. Golm1; PMID: **25816035**) and genes that play a fundamental role in liver fibrogenesis and hepatocellular carcinoma (e.g., Spp1; PMID: [27570486](https://www.ncbi.nlm.nih.gov/pubmed/27570486)). These new data have been added to Table 1, and to lines **379-382** and **523-528**. Second, we searched for genome-wide H3K27me3 datasets for human liver and were able to find *one* study (Hlady et al, Hepatology 2019 PMID:30136421) with data comparing a small number of normal human livers (n=2) to livers from n=4 patients with cirrhotic and HCC livers (sex not specified). In the diseased human livers, loss of H3K27me3 at bivalent promoters was associated with increased transcription of genes associated with tumorigenesis. This finding is consistent with the increased liver disease linked to Ezh1/2 loss in our mouse model. Hlady et al also identified 92 human genes related to liver inflammation that are altered in human cirrhosis, a subset of which are associated with differences in HCC survival (Supplemental table 4). We identified 89 mouse orthologs of the 92 human genes and evaluated their response to Ezh1/2 loss in our model, and found that 19 of these genes are responsive in the DKO, of which 6 are sex-biased genes (5 female-biased genes up-regulated and 1 male biased gene down-regulated). These new findings are added to the manuscript on lines **379-382** and **532-535**.

**Reviewer #2**

Summary
The sexual dimorphic hepatic gene expression profile is linked to the sex-biased incidence and severity of liver diseases, such as liver fibrosis and HCC. This manuscript probed an essential role of EZH1 and EZH2, the catalytic components of the polycomb repressive complex-2 (PRC2), for their regulation of the sex-based repressive chromatin histone mark H3K27me3 and the metabolic and fibrosis gene expressions profile in the liver. This study established that Ezh1/2-based repression of female-biased genes is a major epigenetic mechanism that controls sex-biased gene expression in the male liver and represented a direct demonstration of the functional importance of the sex-biased repressive histone mark H3K27me3. The author provided comprehensive characterization of the hepatic chromatin modification states such as H3K27me3, H3K27ac and H3K4me1, in combination with the previously published datasets from hypophysectomy and continuous GH infusion models. The up regulation of the genes linked to liver fibrosis and HCC is more significant in the male liver but to a lesser extent in the female liver, which may indicate the clinical relevance of Ezh1/2 in patients with liver disease, however, the functional evidence of this observation in vivo is weak. The study design was clear and straight-forward and the manuscript was well polished, but there are some concerns for which additional experiments and revision of the manuscript should be considered.

Author comment: Please note our response to Reviewer 1, comment #2, above, and the new analyses that we have added supporting the relationship between our findings and human liver disease.

Reviewer 2 Major points:

1. Since (i) deletion of Ezh2 in embryonic liver impairs hepatic progenitor expansion, thus the liver differentiation and maturation (ref 25), (ii) Alb-Cre transgene is also active in fetal and neonatal mice (Ref 63), (iii) a more recent study reported that disruption of Ezh1 and Ezh2 in livers caused perinatal hepatocytes to differentiate prematurely (Grindheim et al, Gastroenterology, 156:1834-1848, 2019). [Does] the combined hepatic Ezh1/Ezh2 deficiency model in this study have similar issue? If so, is this defect also sex-specific? And whether the sex-specific differences in the adult mice of current study are secondary to this developmental defect? And how is this defect linked to the context of sex-specific growth hormone regulation?

Author response: We thank the Reviewer for drawing our attention to the recent study of Grindheim et al (2019). First, we established the similarity between our Ezh1/2-DKO mouse model and that used by Grindheim et al, insofar as both models were apparently developed in a very similar manner, and show the same striking effects on sex-specific gene expression in 2-month old mice: female-biased genes were up-regulated and male-biased genes were down-regulated in the absence of Ezh1/2, and there is extensive loss of sex-biased gene expression. Since the Ezh1/2-deficient mouse model used by Grindheim et al is closely related to the Ezh1/Ezh2 DKO model used in our study, we fully expect that the premature maturation of liver genes in Ezh1/2-deficient livers reported by Grindheim et al will also occur in our mouse model.

Next, we re-analyzed the raw RNA-seq reads of Grindheim et al from the perspective of premature maturation of sex-biased genes (which was not done in the analysis reported by Grindheim et al) in order to address the other questions raised by the Reviewer. First, we determined that the set of liver maturation genes, defined as described by Grindheim et al, show a strong enrichment for sex-biased genes. Further, we determined that premature maturation was significantly more extensive in male than in female Ezh1/Ezh2-deficient liver, and that sex-biased maturation genes were significantly enriched in the set of all premature maturation genes, with the strongest enrichments seen in Ezh1/Ezh2-deficient male liver. These analyses suggest, but cannot establish in a definitive manner, that the sex-specific differences seen in the adult DKO mice of the current study are secondary to the developmental defect reported by Grindheim et al. These new findings are presented on lines **392-409**, **660-672**, and in **Table S10,** and **lines 1449-1469**.

2. The author showed the Ezh1/2-dependent and sex-biased regulation of liver fibrosis and HCC-related gene expression in Fig. 7. However, this is largely based on the gene expression analysis, further functional evidence is necessary for the author to draw a solid conclusion. As previous reports mainly used the male mice in their studies (Ref 33 and Grindheim et al, Gastroenterology, 156:1834-1848, 2019), a comparative characterization of the liver fibrosis and damage phenotype between male and female would be informative at this point, either in the older hepatic Ezh1/Ezh2 deficiency mice or in the CCl4-induced hepatotoxity model.

Author response: We have revised the manuscript to add histological data comparing liver pathology of male and female Ezh1/2 DKO mice at 8 months of age, as requested by the Reviewer (**Fig. 7A**). These data indicate greater liver fibrosis and inflammatory cell infiltration in DKO male compared to DKO female mice, whereas the female livers show a more extensive fatty liver phenotype. These new results are added to the text on lines **354-357**.

Minor points:

3. Fig.2 and 3 are very busy - need to be reorganized or streamlined to make them easier to follow.

Author response: We have reorganized the panels in Fig. 2 and moved panel E to Fig. S2, and we edited the proportions of several of the panels in Fig. 3 to make both figures less crowded and easier to follow.

4. Fig.3 - To what extent does EZH1/2-dependnent liver chromatin repression contribute to the GH-based regulation on the sex-based gene expression profile? Please clarify in the text.

Author response:
In Fig. 3C, we elucidated the GH-dependence of E1/E2-KO-responsive female-biased genes by examining a set 113 robust female-biased genes, of which 65 are up-regulated in E1/E2-KO male liver. We determined that 58 of these 65 genes (89%) were up-regulated in liver in response two treatments that disrupt normal circulating GH patterns, hypophysectomy and continuous GH infusion. Moreover, 5 of the 7 genes that were not responsive in the hypophysectomized male or in the continuous GH model, were actually repressed in livers of hypophysectomized female mice. Thus, 63 of 65 (97%) of the E1/E2-KO responsive female biased genes are controlled by GH. This point is now made explicitly in the text (lines **244-247**).

5. Regarding Discussion section (line 503) - the incomplete loss of H3K27me3 marks at many genomic loci, are there any other additional genes or enzymes that could catalyze the H3K27me3 mark formation? Since Alb-Cre transgene is also active in fetal and neonatal mice (Ref 63).

Author response:
To date, EZH1/2 is the only known H3K27 methyltransferase in eukaryotic cells. (Healy et al PMID: **31521505**), as we now note on lines **576-578**.

**Reviewer #3**

This interesting manuscript has utilized Ezh1/Ezh2 hepatocyte double null mice to demonstrate the necessity of H3K27me3 in controlling the sex-specific gene expression that are relevant to liver diseases (such as liver fibrosis and cancer) as well as following toxicant exposure. Comprehensive integrations of RNA-Seq and ChIP-Seq datasets were performed taking the advantage of data generated de novo (the DKO mice) as well as from online data repositories. The authors concluded that the Ezh1/Ezh2 catalyzed H3K27me3 regulates sex dependent genetic programs in liver metabolism and fibrosis through sex-dependent effects on the epigenome and may thereby determine the sex bias in liver disease susceptibility. The findings are novel and important in further understanding the mechanisms of gender specific disease phenotypes.

Major comments:

1) The mouse model used was an Ezh1 whole body knockout crossed with Ezh2 hepatocyte specific knockout mice. Please discuss why Ezh1 hepatocyte specific mouse was not used to cross with the Ezh2 hepatocyte specific knockout mice? Is it because it is embryonically lethal?

Author response: Ezh2 null mutation results in lethality at early stages of mouse development (O’Carroll et.al 2011; PMID: [11390661](https://www.ncbi.nlm.nih.gov/pubmed/11390661)), while Ezh1 null mutation does not decrease survival. Importantly, only the combined loss of Ezh1 and Ezh2 in hepatocytes results in decreases in H3K27me3 mark deposition (Bae et.al 2015). We don’t have information on the hepatocyte specific Ezh1/2 knockout. This information is now added on lines **514-518**.

2) According to NCBI Gene RNA-Seq repository, Ezh1 expression in liver is 3.057 (FPKM), whereas Ezh2 expression in liver is only 0.272 (FPKM). Similar trend of gene expression profiles was also observed in the present study (Figure 1A and 1B). It appears that Ezh2 is barely expressed in liver. This is probably why in DKO there was no difference in the basal expression of Ezh2 as compared to controls. Could extrahepatic depletion of Ezh2 be more important in indirectly modulating the sex-specific gene expression in liver through altering remote hormonal signaling?

Author response: Yes, Ezh2 RNA levels are low in adult liver, but collectively, the expression of (Ezh1 + Ezh2) is both necessary and sufficient for sex-specific deposition of H3K27me3 marks and regulation of the associated sex-biased genes. In addition, genes that show a sex bias in expression in liver are primarily expressed in hepatocytes. Consistent with this, hepatocyte-specific loss of Ezh2, in the context of a global Ezh1-deficient background, dysregulates many sex-specific genes. While a few such genes might be affected by loss of Ezh1/2 in non-parenchymal cells (i.e., non-hepatocytes within the liver) by an indirect mechanism, we do not expect that to be the case in general.

3) Figure 1A: it is not clear exactly how the data were expressed for RT-qPCR data. Were they normalized to housekeeping gene and expressed as % of housekeeping genes? Also it will be helpful to include the Cq values.

Author response: Raw Ct values were analyzed using the comparative Ct method with normalization to the 18S RNA content of each sample, as explained on **line 607**. Shown in the revised Fig. 1A are the relative expression levels, with the 8 week old male liver group set to a relative expression level of 1.0. We have added the average Ct values for each qPCR group on top of each bar.

4) Figure 2. It is very interesting that hepatic Ezh1/Ezh2 deficiency down-regulates many male biased genes, and the authors suggested that this was due to a secondary response to the disruption of female-biased gene expression. However, it will be even more interesting to conduct mechanistic investigations regarding how this secondary response occurs. e.g. is there any enhanced DNA methylation at these gene loci? Also it will be interesting to conduct up-stream regulator analysis at these down-regulated genes and see what factors may serve as repressors of these genes in the absence of H3K27Me3 enzymes.

Author response: First, we observed a loss of a large fraction of the male-biased activating marks (H3K27ac and H3K4me1) in DKO male liver (Fig. 6B), which we previously associated with male-biased genes (Sugathan and Waxman, MCB 2013), consistent with the down-regulation of many male biased genes in DKO male liver. We did not analyze the DKO livers for any effects on DNA methylation, but will consider such work in the future. Second, we have now carried out IPA Upstream Regulator analysis, which provided several interesting insights, now presented on lines **189-204, 464-466** and **548-551**.

5) The authors focused on liver fibrosis and HCC pathways in Figure 7. However, in Figure 2, it would be interesting to know in general what pathways (e.g. KEGG) are affected using a top-down approach.

Author response: We have now carried out DAVID analysis, including KEGG pathway analysis of the genes shown in Figure 2, and our findings have been added on lines **147-151**.

6) It is very interesting that Ezh1/Ezh2 deficiency increased Cyp2b9 mRNA in males, but did not increase Fmo3 mRNA in males; however, in Fmo3 gene loci, H3K27me3 was also markedly decreased in DKO males (similar to Cyp2b9). This indicates other suppressive marks are present – it would be interesting to explore this.

Author response: We think it most likely that loss of repressive marks alone is not sufficient to de-repress Fmo3 and other such genes; likely those genes need to acquire activating marks at key enhancers in order for them to be de-repressed, and as such, loss of Ezh1/2 alone in not sufficient to reverse the impact of ongoing GH signaling that does not support gene activation. This issue is discussed more fully on lines **4531-458**.

Reviewer 3 Minor comments:

1) I suggest changing “sex-biased genes” to “sex-enriched” or “sex-specific” genes. Biased usually refers to opinions.

Author response: We fully understand the reviewer’s suggestion, and have now added in the term “sex-enriched” on line 66-67 in addition to ‘sex-biased’. It may be noted that the term sex-biased has emerged as a preferred term used by many in this context, including in the title of a recent Annual Reviews article: “Sex-Biased Gene Expression”. Grath S et al. Annu Rev Genet. (2016). We do not use the term sex-specific, as that implies a gene is specifically (i.e., exclusively) expressed in one sex, not the other. This is not factually correctly for a majority of the genes we designate as sex-biased, insofar as in many cases gene expression differs by only 2-4 fold; hence expression is not specific (exclusive) to one sex.

2) CCl4 is not a hepatotoxin, it is a hepatotoxicant.

Author response: We have retained the term ‘hepatotoxin’, as a PubMed search indicates it is used four times more frequently than ‘hepatotoxicant’ in the context of carbon tetrachloride (n=212 vs 53 entries). Also, Miriam Webster Medical Dictionary defines *hepatotoxin* as “a substance toxic to the liver”, but for *hepatotoxicant*, the dictionary reports: *The word you've entered isn't in the dictionary.*

**Have all data underlying the figures and results presented in the manuscript been provided?**
Large-scale datasets should be made available via a public repository as described in the *PLOS Genetics* [data availability policy](http://journals.plos.org/plosgenetics/s/data-availability), and numerical data that underlies graphs or summary statistics should be provided in spreadsheet form as supporting information.

Reviewer #1: Yes
Reviewer #2: Yes
Reviewer #3: None

Author response: We have now provided the missing information requested by Reviewer #3. Further, we have provided several new Supplemental tables to provide a more complete set of underlying data supporting our analyses.
